# Miscellanies

**Published:** 1844-01-01

**Authors:** 


### Miscellanies.

---

Medical Statistics of the United States Frigate, Potomac, Commodore John Downes, Commander, during a Three Years' Voyage of Circumnavigation of the Globe. By J. M. Foltz, Esq. Surgeon, U. S. Navy.

The above frigate, Potomac, 44, with a complement of 500 men, performed a voyage of circumnavigation of the globe, during a cruise of three years. During this period she traversed 97 degrees of latitude, from 40° N. to 57° S. crossing the Equator six times. A period of nearly two years was passed between the Tropics; and it may be here added, as singular, that while three months were spent within 6° of the Equator, in the East Indies, where a malignant dysentery appeared on board, the same period, subsequently, was passed in the same latitude at the direct antipodes, in the vicinity of the Gallapagos Islands, where, from the absence of marshes, rank vegetation, and consequently of malaria, the crew enjoyed the average health of our own latitudes. In this paper, Dr. Foltz gives a concise statistical account of the health of the crew, with a brief notice of the character of the diseases developed in the different climates traversed, as well as those resulting from confinement on board-ship, with observations on the medical topography of the most important parts visited.

On the 24th of Aug. 1831, the Potomac left the harbour of New York, with 502 souls on board, all in apparent good health. On the 6th of October crossed the Equator, thermometer at 79°, with fresh trade winds and cloudy weather—28men on the sick report—53 days after leaving New York arrived at Rio, 22° 30′ South lat. This place, notwithstanding its inter-tropical location, is free from malignant diseases—cutaneous diseases are very prevalent. On the 5th of November sailed for the Cape of Good Hope, and anchored in Table Bay on the 6th of December—passage boisterous—much rain and thick foggy weather—thermometer averaged 60° at noon throughout the passage. Forty attacked with dysentery and diarrhœa. On the 12th of December, sailed from Table Bay. After doubling the Cape, had a rough sea—cold, wet weather, with westerly winds. Several cases of pleuritis occurred, and also catarrh and rheumatism. Arrived at the Island of St. Paul's, thence directed course to Quallah Battoo, on the west coast of Sumatra, and arrived there on the 5th of February; remained here 12 days. The sick list, which consisted only of 3, now increased to 57—52 cases of disease of the bowels and 12 of bilious fever were treated within the month, notwithstanding every precaution was used to protect the men. The Malays in this vicinity are very healthy—the numerous merchant vessels which visit this coast, enjoy comparative health, when on the Coast of Sumatra, while the shores of Java and Borneo are shunned as a pest-house. Feb. 16. Sailed from the West coast of Sumatra and crossed the Line on the 20th, with thermometer at 85°. On the 25th, sick list reduced to 30. On the 28th anchored at Bantam Bay, on the north-western extremity of Java. A change was made here in the rations—rice and curry were substituted for beef, pork, and beans. The spirit ration was divided into three portions, one given in the morning, one at noon, and the other in the evening—all hands were ordered to wear flannel. Remained here 12 days; during this period mean temp. at noon 82°. Fifteen cases of dysentery and four of fever admitted, with a daily average of 29 sick during our stay. 19th of March, arrived at Batavia—this port has been more fatal to navigators than any other on the globe. It was in this port that Dr. James Johnson encountered the malignant fever which committed such dreadful ravages in the English squadron in 1806, and which forms the basis of his invaluable work upon the diseases of tropical climates. Every precaution in the way of prevention was adopted. To Dr. Johnson we were much indebted for many valuable hints in prevention, indeed to him we acknowledge professional obligations, which it will be ever out of our power to repay. But little variation in the temperature during our stay at Batavia—the mean at noon was 82°—the land breeze which reached us in the evening was charged with the most offensive effluvia from the fens and marshes. Notwithstanding all our precautions, the number of sick daily increased, the prevailing disease being a severe form of dysentery, accompanied with much inflammation, tormina and tenesmus. Copious bloodletting, local and general, at first—emetics to counteract the great tendency to visceral congestion, and to restore a healthy action to the deranged functions of the skin and liver. Mercurials, with opium and ipecacuanha, were found most beneficial—mercurial inunctions were also used to accelerate the action of the mercury—ptyalism always gave relief. When this treatment did not arrest the progress of the disease within a few days, bloody, fætid evacuations followed, with gangrene, collapse, and death. April 10th. Sailed from Batavia with 42 cases of dysentery—on the 20th, crossed the Equator, therm. at 90°, number of sick increasing—chloride of lime was used freely about the cots. After a passage of 39 days we arrived at Canton. From this time the health of the crew improved. June 5th. Sailed for the Sandwich Islands—the occurrence of strong westerly winds and cool wet weather soon changed the character of the diseases on board, and pleuritis, intermittents and inflammatory affections took the place of dysentery—25 average on the sick report. July 23. Arrived at the Sandwich Island, Oahee. The natives here suffer much from a species of leprosy, called the *craw-craws;* to cure which they have recourse to the *Kava-root,* a powerful alterative and narcotic. Left on the 15th Aug. and in 28 days arrived at Otaheite. On our passage from this to Valparaiso, it being the winter here, we had cold weather, wind and rain, which again changed the type of disease—15 cases of pneumonia and pleuritis, with 22 of rheumatism. In 34 days, arrived at Valparaiso with 36 sick. Dec. 2. Sailed for Lima, and arrived at Callao on the 15th, the sick report being reduced to 18—remained in this port 75 days—the weather here uniformly clear with a delightful south wind—here it never rains—dysentery sometimes prevails to a great extent, and during July and August, those predisposed to tubercular phthisis are liable to have the disease developed. Feb. 28. Sailed for Valparaiso, and reached it in 16 days. April 25, a case of small-pox occurred, and in a few days another case, followed by another—those were sent on shore—other cases occurring, made it evident that the contagion was on board-ship. It was therefore determined to inoculate the whole ships' company. 287 were inoculated—85 of those inoculated took the disease, many of them having it in a mild form. Next sailed for Callao, and then for the Gallapagos Islands, and, Sep. 1, anchored in Essex Bay, Charles Island—remained 10 days in port, the mean of the therm. being 73°, that of barom. 20.90 in. and the average on sick report, including several cases of dysentery, was 21. All those attacked with dysentery, had suffered from the same disease at Batavia, the Antipodes of our present position. Hence sailed to Guayaquil, and arrived at Puna. Here the crew were much disposed to disease of the intestinal canal—dysentery continued to increase ; it was not so malignant or intractable, however, as at Batavia. Some cases of chronic hepatitis also occurred, which yielded to mercurial alteratives and the nitro-muriatic acid, internally and in baths. Sept. 28. Sailed for Payta. The cruising-grounds for the American ships employed in the sperm whale fisheries are directly off this port; they frequently resort here for fruits and vegetables, as antiscorbutics. At the head of the list of the anti-scorbutics among the whalers stand raw potatoes ; two are served out daily to each man, and eaten raw with vinegar. On the 9th of February, sailed for the United States from Valparaiso. March 6, were off Cape Horn. Character of the diseases now changed. Cases of pleuritis, cynanche tonsillaris, and rheumatism, augmented the sick report. On the 23d of March arrived at Rio—remained here 16 days. After leaving Rio several cases of diarrhœa occurred. April 27, crossed the Equator, and reached Boston 23d May. On our arrival at the United States, the health of the crew was such as to enable all but six to take their discharge. The Potomac, during the voyage of circumnavigation, sailed over 61,000 miles, having been at sea 514 days. She crossed the Equator six times, varying from 40° North to 57° South.

---

### The Endemic Influence of Civil Government, illustrated in a View of the Climate, Topography, and Diseases of the Island of Minorca, with an Account of its Medical Faculty—of the French Military Hospital on the Isle de los Reyos—and of the Statistics of the United States Naval Hospital at Mahon, for the Years 1839-40 and 41. By J. M. Foltz, A.M., M.D., Surgeon U.S. Navy.

We shall extract from this article those points which appear the most interesting to the medical reader. Dr. Foltz, the writer, served as surgeon to the Naval Hospital established in Minorca, this island having been the depôt for the United States' naval forces in the Mediterranean for more than twenty years. The period of the writer's residence in the place was from 1839 to 1841 inclusive. With respect to the *diseases* of Minorca, he observes that their history, as given by Cleghorn, who was stationed in this place as Port-surgeon from the year 1744 to 1749, at a time when the island was in the hands of the British, exhibits, as compared with the present, a complete revolution in their character within the century; this change he accounts for by the pernicious influence of bad government, since the place has fallen into the possession of the Spaniards.

In Cleghorn's time the population of the Island was 40,000, and such was the healthy state of the place, that, in a residence of five years, this writer states, that he did not "meet with a single individual who was lame or deformed," and "cases of paralysis were of extremely rare occurrence." At present the population is reduced to 18,000, and the streets and highways are crowded with the maimed and the blind, whilst the number of mendicants and the poverty of the natives is now so great, that there is no hospital for them. *Fevers* are of considerable frequency; they are almost invariably of a mild tertian form—the cold stage is very short; the hot stage much more violent, and accompanied usually with delirium; this is followed by an intense sweating stage, which occasions great prostration and emaciation, and renders the patient very susceptible to organic disease on the slightest exposure. The following prescription was found very effectual in the treatment of the most obstinate intermittents, both in America and Minorca, in the hands of Dr. Foltz.

℞. Pulv. Cort. Cinchonæ . . . ℥j.

Bitartrat. Potassæ . . . . ʒij.

Pulv. Caryophyll. . . . . ʒj.

Vin. Rubr. (Port.) . . . . Oj. M.

Dose—one ounce to be taken every hour, for six hours preceding the expected paroxysm.

*Neuroses.*—Next in frequency, but first in point of fatality, stand the diseases of the nervous system; they are to be found here from the most insidious forms of neuralgia to the aggravated modifications of paralysis and mania. *Cancerous affections,* including *lupus,* as also *caries* and *necrosis,* are very numerous; they are confined, however, to the poor. *Aneurysm* is a very common disease, and generally proves rapidly fatal, from the circumstance of its proper treatment not being understood. It was to a case of aneurysm that Dr. Foltz was indebted for his introduction to his medical confrères at Mahon.

The Medical Faculty of Minorca is under the direction of the Royal Medical Junto at Madrid, without whose license no one is permitted to practise medicine or surgery within the dominions of her Catholic Majesty. The medical sub-delegate for Mahon is Dr. F. Fernandez, a well-educated and clever practitioner; there are other practitioners also of respectable professional attainments; but they are all eclipsed by a host of illiterate, ignorant pretenders, who purchase by a bribe a license to practise—at the head of these is a man of much tact, and great effrontery combined with great moral courage, who is ever ready to rush into every operation, generally to the ruin of his patient; still he monopolizes the most lucrative practice of the place. This man was originally a barber. The medicines most in repute for every form of disease are Morrison's pills, and Leroy's purgative mixture. Dr. Foltz witnessed several fatal results in cases in which they were employed in Mahon. The Doctor and his medical friends who accompanied him, were frequently consulted, and requested to perform several operations, which they did. In this way they learned much regarding the state of the profession, and the various modes of treatment; among these, charms, amulets, and the superstitious influence of the Church occupied a large share. The priest sells amulets to ward off fever; keeping a cross, they say, from the fig-tree, over the heart, will have the same effect. He quotes several instances of the disgusting and ridiculous mode of treatment employed here. The practice of the Doctor and his countrymen being wholly gratuitous, soon became extensive; the native practitioners, however, became jealous, and soon put a stop to them. The only hospital at Mahon is an hospital for foundlings, which is liberally endowed. There is not in the Island a dispensary for the distribution of medicines to the poor; and very little charity from the medical men, who are, indeed, compelled by their own wants to seek some remuneration for their services.

Near the centre of the harbour of Mahon is the Isle de los Reyos, a small island on which a large naval hospital was erected in 1796, for the use of the British fleet. In the Summer of 1839, Dr. Foltz visited Algiers, the first year of the war against the Bedouin chief. Here he ascertained that there was an average of three thousand deaths among the troops from dysentery and fever, since the commencement of the war. The total number sick in the hospital at Algiers at the period of his visit was near three thousand. The French Government applied for the use of the hospital at Mahon, for such of the sick as could be transported there—the application was granted. The medical part of the Hospital was under the charge of M. Hutin, and the surgical under that of M. Jourdan, to whom were attached thirty medical assistants. The treatment was decidedly Broussaian. Dr. Foltz, who had himself encountered this disease in the East Indies, where its progress was so rapid that it often proved fatal within 48 or 72 hours, found that the only safety for the patient consisted in an energetic and vigorous treatment. This disease in Africa presented many of the peculiar symptoms met with in the disease as it occurred in India, particularly in the derangements of the functions of the skin and liver, in which the prompt use of the lancet and mercury were loudly demanded. For a knowledge of the judicious and successful treatment of this disease he acknowledges the medical practitioner to be indebted to the excellent work of Dr. James Johnson on Tropical Climates; he further states, that much esteemed as this work is in Europe and the United States, it is to the practitioner in inter-tropical climates chiefly that its great merits can be known. Dr. Foltz next proceeds to give an account of the United States' Naval Hospital at Mahon.

The majority of admissions into this hospital were from diseases of the intestinal canal; this tendency to gastric disease, especially in hot climates, is doubtless produced by the indigestible diet to which they are confined at sea. He found the most careful treatment of little avail, as long as the patient was confined on board-ship. When ulceration had taken place in the mucous membrane, as happens in a majority of cases, he found the mineral astringents the most effectual; and at the head of these he places the acetate of lead, in doses of from one to two grains, twice a day, in combination with one-eighth of a grain of opium. The sulphate of copper also, combined with opium or lactucarium, he also used with benefit; but a careful attention to diet will be found most important in such cases, to which the use of enemata of cold water, or a simple infusion of sem. lini are useful adjuvants. The occurrence of delirium tremens is very frequent among seamen, as may naturally be expected. The treatment adopted by Dr. Foltz, and found most effectual, were emetics and opium, and, where much arterial excitement existed, venæsection. During an experience of more than twelve years in the Navy, only one fatal case of this disease came under his observation, and in this instance it was associated with hæmoptysis.

He is satisfied that, notwithstanding the lauded salubrity of the Mediterranean, and the advantages it holds out to the pulmonary invalid, Minorca at least possesses none of these recommendations. On the contrary, he found that all who were predisposed to pulmonary disease, or were of a tubercular diathesis, were sure to have the latent disease developed. Dr. Foltz, having met with an article in the Medico-Chirurgical Review, on the advantages of high temperature after injuries and important surgical operations, determined to give the plan of treatment a fair trial. An opportunity presented itself here in the case of a man, who had the upper and posterior portion of the right lung severely wounded by a stab of a knife. The temperature of the apartment, in which this patient lay, was kept as high as the patient could endure without inconvenience. This patient recovered, which the Doctor attributed, in a great degree, to the high temperature. We feel it necessary to discontinue our notice of this paper from want of space.—*New York Journal of Medicine,* July, 1843.

---

### An Opposition Coach.

Priessnitz has got a rival close to his own castle! About four miles from Graeffenberg, in the lovely valley of Lindiviesse, Dr. Schrott, an old school-fellow of the Apostle of Hydropathy, has started a Cure-all, in opposition to the Silesian peasant. His Methodus Medendi is no vile imitation of that of the far-famed Practitioner of Graeffenberg. It is a veritable Antipous. The one inculcates the drinking of water, and water only. Schrott, forbids water and all other fluids—and thus cures by thirst! The following extract from Mr. Beamish, one of the disciples of Priessnitz in this country, will give a notion of the New Dipsopathic doctrine and practice.

"About four miles from Graeffenberg, up a lovely valley, is situated the village of Lindiviesse, where dwells a school-fellow of Priessnitz, by name Schrott, a remarkable, but illiterate man, who told me that he had never opened a book on medicine, physiology, or anatomy, and that he never would. He undertakes to cure all diseases, not by the exhibition of cold water, which *he ridicules,* but by withholding from his patients all fluids. The treatment which he has adopted, and which may be termed the Dipsopathic (,Διψα, thirst), though clearly applicable to a variety of ailments as we shall presently show, I found, in a long interview, to emanate from strangely confused physiological notions. He talked of placing the human being in the same condition as it existed in the womb, by means of moist warmth, with which he surrounds it, (the *feuchte wärme,*) communicated by *three* wet sheets,, in which the patient sleeps. They are all applied in a manner similar to Priessnitz's *one,* the process usually commencing at two o'clock in the morning. The patient remains packed up till eight or ten o'clock. The system of total abstinence from drink is carried on for *five,* and sometimes for *eight* days consecutively; the alvine excretions cease, and the urine is excreted in small quantity, very turbid, and deposits various salts. One patient told me that he had been twelve days without any relief from the bowels; and I heard also of one who had been *seven weeks*. Sometimes, however, diarrhœa occurs, which Schrott considers as a favourable crisis.

"By depriving the stomach of fluid, the absorbents of the skin are brought into powerful activity; and, by the moisture having to travel from the extremities of the frame, Schrott thinks that it carries with it the humours of the blood to the bladder, from whence they are ultimately expelled with the urine; because in the urine he finds large deposits, to which he triumphantly points as containing the extraneous matter that caused the disease." 98.

Mr. Beamish, while he protests against this hunger and thirst cure, as invading the territory of the water-cure, candidly acknowledges that there may be cases that have resisted the Allopathic and even the Hydropathic Systems, and which might be treated with success by the Dipsopathic plan. "I allude to those where there had been serous or sanguineous effusion, or *dilatation* of part of the brain." We need not say more.

---

